# Highly Divergent SARS-CoV-2 Alpha Variant in Chronically Infected Immunocompromised Person

**DOI:** 10.3201/eid2809.220875

**Published:** 2022-09

**Authors:** Bas B. Oude Munnink, Roel H.T. Nijhuis, Nathalie Worp, Marjan Boter, Babette Weller, Babs E. Verstrepen, Corine GeurtsvanKessel, Maarten F. Corsten, Anne Russcher, Marion Koopmans

**Affiliations:** Erasmus Medical Center, Rotterdam, the Netherlands (B.B. Oude Munnink, N. Worp, M. Boter, B. Weller, B.E. Verstrepen, C. GeurtsvanKessel, M. Koopmans);; Meander Medical Center, Amersfoort, the Netherlands (R.H.T. Nijhuis, M.F. Corsten, A. Russcher)

**Keywords:** COVID-19, Alpha variant, virus evolution, immunocompromised, variants, coronavirus disease, SARS-CoV-2, severe acute respiratory syndrome coronavirus 2, viruses, respiratory infections, zoonoses, the Netherlands

## Abstract

We detected a highly divergent SARS-CoV-2 Alpha variant in an immunocompromised person several months after the latest detection of the Alpha variant in the Netherlands. The patient was infected for 42 weeks despite several treatment regimens and disappearance of most clinical symptoms. We identified several potential immune escape mutations in the spike protein.

Persons with an immune deficiency can be infected with viral pathogens for a prolonged period. This occurrence has been reported for noroviruses (*1*) but also has been documented for SARS-CoV-2 (*2*). We report a patient with type 2 diabetes mellitus and chronic lymphocytic leukemia who had been infected for 42 weeks with SARS-CoV-2. The patient was hospitalized on April 23, 2021, and received optiflow treatment with dexamethasone, tocilizumab, and remdesivir. After May 11, 2021, the patient recovered and experienced no residual symptoms. Almost 9 months later, on February 3, 2022, the patient was readmitted to the hospital for leukemia-related anemia and tested positive for SARS-CoV-2 once again. A month later, the patient died of causes unrelated to SARS-CoV-2.

In all, the patient tested positive for SARS-CoV-2 in 6 nasal or pharyngeal swab specimens. We performed whole-genome sequencing on all specimens by using an amplicon-based sequencing approach, as previously described, with the updated ARTIC primers version 4.1 (ARTIC Network, https://artic.network/mcov-2019) (*3*). The sequencing was successful for 2 specimens from mid-2021 and 2 specimens from early 2022 (Table). Pangolin version 4.0.6 PLEARN-v1.8 classification using default settings demonstrated that all sequenced viruses belonged to the Alpha (B.1.1.7) variant of concern (VOC) (*5*), Nextclade version 1.14.1 strain 20I (*6*). The samples were run on flowcells containing 96 samples, including a positive and negative control (pangolin lineage B.1.77.50) to exclude potential contamination. GISAID’s EpiCoV database (https://www.gisaid.org) showed that the latest isolate identified as Alpha in the Netherlands was collected on October 13, 2021, suggesting that the variant had not been circulating in the Netherlands since that time. Phylogenetic analysis by IQ-TREE (*7*) using all unique downsampled Alpha sequences available in GISAID (*8*) from the Netherlands showed that the viruses detected on January 31 and February 3, 2022, were identical but distinct from previously observed Alpha lineages in the Netherlands (Figure). A zoom-in of the phylogenetic tree showed that all sequences of the virus in the patient cluster together in a separate branch, suggesting that the patient was chronically infected with this specific variant of SARS-CoV-2 (Appendix Figure 1).

**Table:** Mutations detected by WGS in an immunocompromised person chronically infected with highly divergent SARS-CoV-2 Alpha variant in the Netherlands compared with Wuhan-Hu-1 variant (GenBank accession no. NC_045512.2)*

Collection date	GISAID (ENA) accession no.	Ct value†	ORF1ab mutations	Spike mutations	E gene mutations	N gene mutations
2021 Apr 13	EPI_ISL_10688798 (ERS1216848)	20.3				
2021 Apr 28	NA	17.9	NS	NS	NS	NS
2021 May 5	NA	22.3	NS	NS	NS	NS
2021 May 26	EPI_ISL_2887843 (ERS7202253)	22.1	C5178A, G5880T, C12852T, C17555A	NM	NM	NM
2022 Jan 31	EPI_ISL_10072285 (ERS10975083)	NA‡	A570T, G713T, G4232T, C5178A, G5880T, C11653T, C11665T, C12528T, A12555G, C12624T, G12761T, C12774T, C12852T, C17555A, T18340C, G20578T	T21752A, G21987T, G22578A, C22879A, G23009T, C25282T, C25350T	T26418C	NM
2022 Feb 3	EPI_ISL_13133128 (ERS11204596)	23.6	A570T, G713T, G4232T, C5178A, G5880T, C11653T, C11665T, C12528T, A12555G, C12624T, G12761T, C12774T, C12852T, C17555A, T18340C, G20578T	T21752A, G21987T, G22578A, C22879A, G23009T, C25282T, C25350T	T26418C	NM

*Wuhan-Hu-1 strain, GenBank accession no. NC_045512.2. Ct, cycle threshold; E, envelope; ENA, European Nucleotide Association (https://www.ebi.ac.uk/ena/browser); GISAID, https://www.gisaid.org; ORF1ab, open reading frame 1ab; N, nucleocapsid; NA, not applicable; NM, no mutations compared with the sequence from the earliest timepoint; NS, whole-genome sequencing not successful; WGS, whole-genome sequencing. †Real-time PCR as described previously was performed (*4*) to test for the E gene and the RNA-dependent RNA polymerase gene. The lowest Ct value is reported. ‡This specimen was tested using the Aptima SARS-CoV-2 assay (Hologic, https://www.hologic.com) using the Panther system. This system does not provide Ct values.

**Figure:**
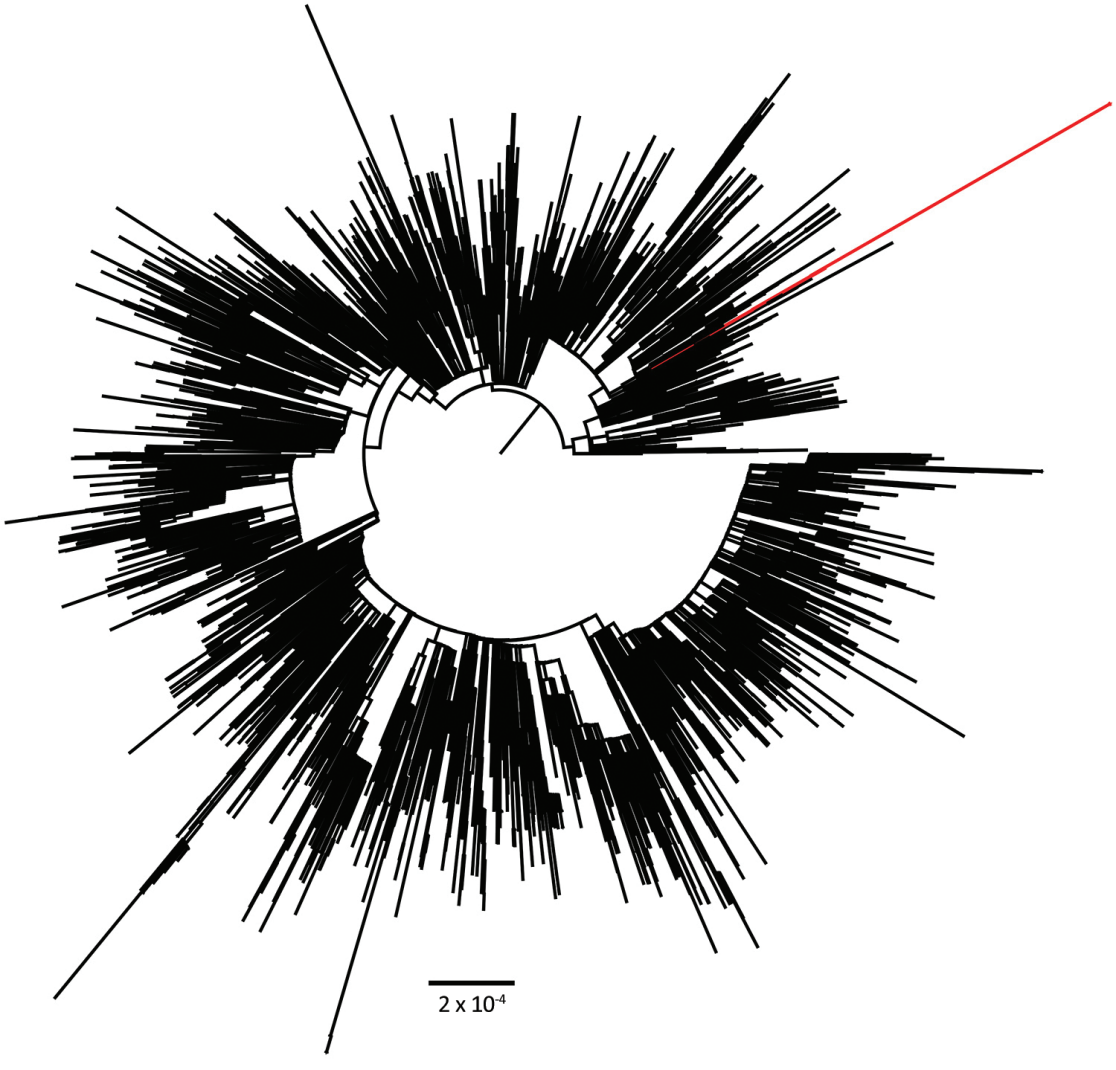
Phylogenetic analysis of all downsampled SARS-CoV-2 Alpha variants (B.1.1.7) in the Netherlands. Scale bar indicates number of substitutions per site.

Over time, we identified 24 nucleotide mutations when we compared sequences from the earliest and latest timepoints. Of these mutations, 19 mutations were nonsynonymous, resulting in 13 amino acid mutations in open reading frame 1ab and 6 amino acid mutations in the spike protein (Table; Appendix Figure 2). Of the 6 mutations in the spike protein, 3 are located in the receptor-binding domain (G339D, N439K, and V483F), 2 are located in the N-terminal domain (W64R and G142V), and 1 is located in the transmembrane domain (P1263L). The mutation G339D can also be found in all Omicron VOCs. G142V has coevolved independently in >1 immunocompromised person with a long-term Alpha variant infection (S.A.J. Wilkinson et al., unpub. data, https://doi.org/10.1101/2022.03.02.22271697), and a mutation in the same position (G142D) has also been described in the Delta (B.1.617.2) and in all Omicron variants.

Our data imply that, despite receiving treatment with dexamethasone, tocilizumab, and remdesivir and being discharged without residual symptoms, the patient had not cleared the infection. Unfortunately, ex vivo rescuing of the viruses from the swabs to assess potential immune escape from circulating neutralizing antibodies was not successful, but some of the mutations we observed in this immunocompromised person with long-term SARS-CoV-2 infection could be linked to immune escape. Previous studies suggest that the G339D mutation affects neutralization in a subset of neutralizing antibodies (*9*) and that the N439K mutation causes immune escape and enhances binding affinity for human angiotensin-converting enzyme 2 (*10*,*11*). In addition, the V483F mutation has been shown previously to cause immune escape (*12*).

The constellation of this particular set of mutations has not been found elsewhere yet despite active ongoing genomic surveillance, which indicates the virus did not spread in the population (Appendix Table). Nonetheless, the detection of an Alpha variant infection in an immunocompromised person in a time when Omicron was the primary circulating variant indicates that reinfection is unlikely, which is also supported by phylogenetic analysis. This occurrence illustrates that this VOC did not completely disappear although it was last detected on October 13, 2021, in the Netherlands. In addition, several mutations were found that are also present in other VOCs, suggesting that VOCs might have emerged in long-term infected immunocompromised persons as suggested previously (*13*).

Our findings illustrate that in previously unidentified reservoirs, such as immunocompromised persons, virus variants might still be present even when these variants are regarded as extinct and are no longer circulating among the population. In addition, we show that several mutations associated with immune escape that maintain virulence and fitness can accumulate in such an immunocompromised person. Continuous genomic surveillance in long-term infected persons is essential to elucidate their potential role in the emergence of future VOCs. 

Appendix Additional information about highly divergent SARS-CoV-2 Alpha variant in chronically infected immunocompromised person.
