# Supplementary material for: Highly Divergent SARS-CoV-2 Alpha Variant in Chronically Infected Immunocompromised Person
Source: Emerg Infect Dis. 2022 Sep;28(9):1920–3. doi: 10.3201/eid2809.220875 (PMC9423911; doi:10.3201/eid2809.220875)
Supplement: Appendix — Additional information about highly divergent SARS-CoV-2 Alpha variant in chronically infected immunocompromised person. [file 22-0875-Techapp-s1.pdf]

Article DOI: https://doi.org/10.3201/eid2809.220875

Highly Divergent SARS-CoV-2 Alpha Variant in Chronically Infected Immunocompromised Person

Appendix

Appendix Table. Mutations overview in the spike protein and prevalence worldwide in Alpha variant viruses submitted to GISAID before May 30, 2022

Mutation	Found in the Netherlands	Found worldwide
Spike: W64R	1×	352×
Spike: G142V	38×	2,902×
Spike: G339D	-	81×
Spike: N439K	-	89×
Spike: V483F	24×	399×
Spike: P1263L	7×	1,176×

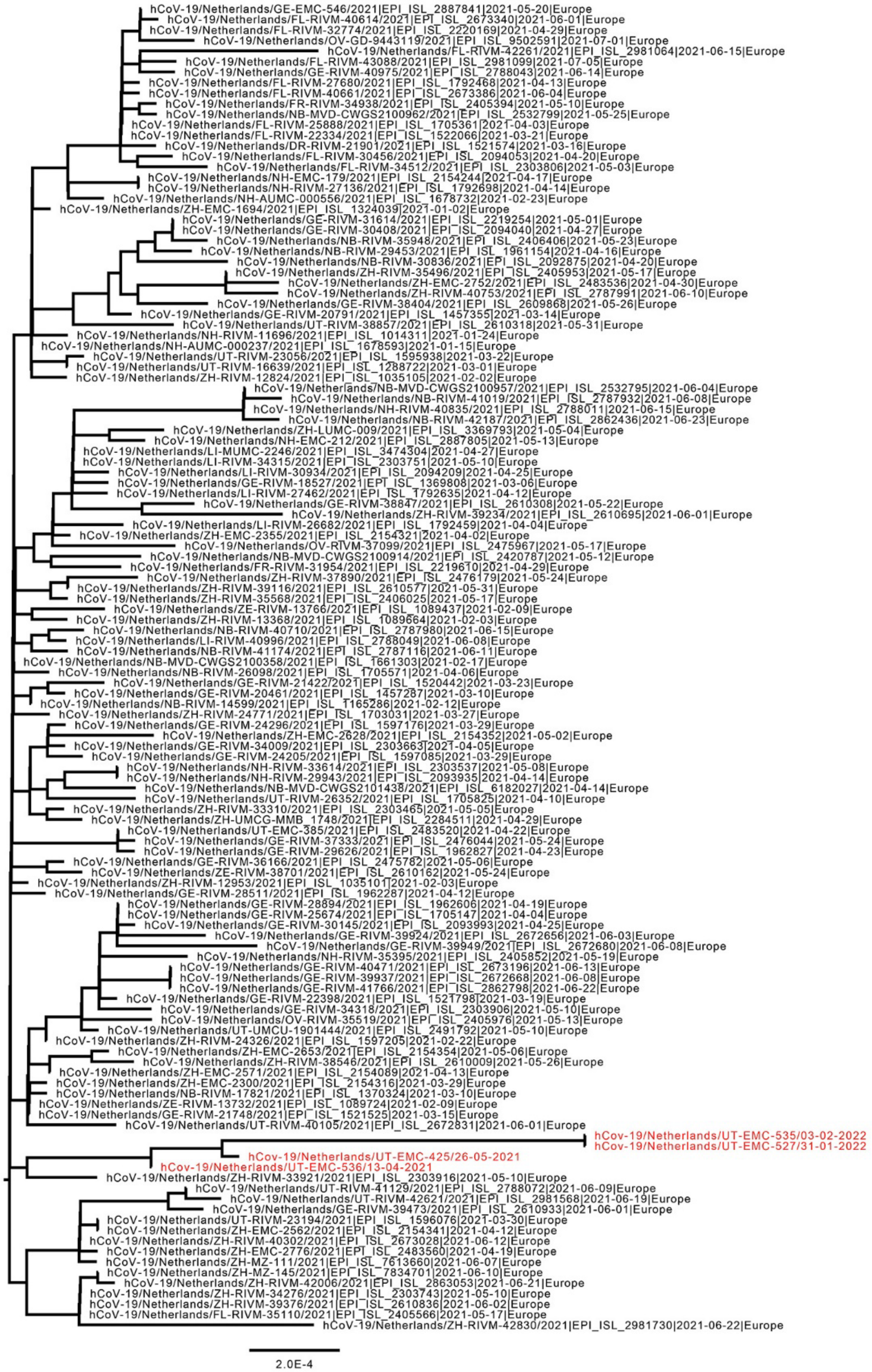

Appendix Figure 1. Zoom of the phylogenetic analysis of all downsampled SARS-CoV-2 Alpha variants (B.1.1.7) in the Netherlands. The sequences from the same long-term infected person are depicted in red. Scale bar indicates number of substitutions per site.

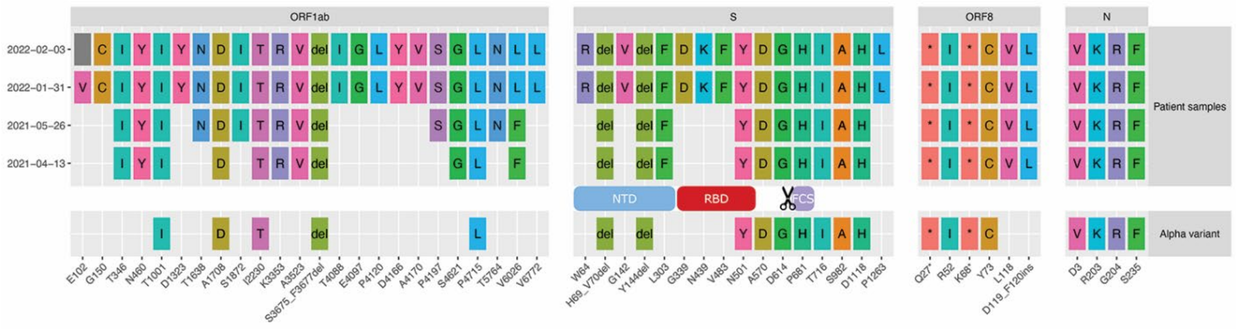

Appendix Figure 2. Amino acid substitutions, deletions, and insertions over time. Mutations are compared to Wuhan-Hu-1 (NC_045512.2). FCS, furin cleavage site; NTD, N terminal domain; RBD, receptor-binding domain.
